# Mesenchymal Stem Cell Exosome and Fibrin Sealant Composite Enhances Rabbit Anterior Cruciate Ligament Repair

**DOI:** 10.1177/03635465241313142

**Published:** 2025-02-21

**Authors:** Keng Lin Wong, Kristeen Ye Wen Teo, Gin Way Law, Shipin Zhang, Tianqi Wang, Hassan Afizah, Chee Jian Pua, Barry Wei Loong Tan, James Hoi Po Hui, Wei Seong Toh

**Affiliations:** * Department of Orthopaedic Surgery, Yong Loo Lin School of Medicine, National University of Singapore, Singapore; † Department of Orthopaedic Surgery, Sengkang General Hospital, Singhealth, Singapore; ‡ Department of Orthopaedic Surgery, National University Hospital, Singapore; § Tissue Engineering Programme, Life Sciences Institute, National University of Singapore, Singapore; ‖ National Heart Research Institute of Singapore, National Heart Centre Singapore, Singapore; ¶ Faculty of Dentistry, National University of Singapore, Singapore; # Integrative Sciences and Engineering Programme, NUS Graduate School, National University of Singapore, Singapore; Investigation performed at National University of Singapore, Singapore

**Keywords:** mesenchymal stem/stromal cells, extracellular vesicles, exosomes, anterior cruciate ligament, repair

## Abstract

**Background:**

The anterior cruciate ligament (ACL) fails to heal after rupture, leading to joint instability and an increased risk of osteoarthritis. Mesenchymal stem/stromal cell (MSC) exosomes have reported wide-ranging therapeutic efficacy; however, their potential for augmenting ACL repair remains to be investigated.

**Purpose:**

To evaluate the use of MSC exosomes with fibrin sealant on biological augmentation of ACL healing after suture repair and their effects on ACL fibroblast functions.

**Study Design:**

Controlled laboratory study.

**Methods:**

Twelve rabbit knees underwent ACL transection and suture repair. MSC exosome and fibrin composite (Exosome+Fibrin) or fibrin (Fibrin) alone was used to supplement the suture repair in 6 knees. ACL repair was assessed by magnetic resonance imaging at 6 and 12 weeks postoperatively and by histologic and immunohistochemical analyses at 12 weeks. To investigate the mechanisms through which MSC exosomes augment ACL repair, metabolic activity, proliferation, migration, and matrix synthesis assays were performed using the primary ACL fibroblasts. RNA sequencing was also performed to assess global gene expression changes in exosome-treated ACL fibroblasts.

**Results:**

Based on magnetic resonance imaging findings, 5 of 6 Exosome+Fibrin–treated ACLs were completely or partially healed, as opposed to 5 of 6 Fibrin-treated ACLs appearing torn at 6 and 12 weeks postoperatively. Additionally, 4 of 6 Exosome+Fibrin–treated ACLs were isointense, as compared with 5 of 6 Fibrin-treated ACLs that were hyperintense, indicating improved remodeling and maturation of the repaired ACLs with Exosome+Fibrin treatment. Histologically, Exosome+Fibrin–treated ACLs showed more organized collagen fibers and abundant collagen deposition, with a high amount of collagen I and relatively lower amount of collagen III, which are consistent with the matrix structure and composition of the normal ACL. Cell culture studies using ACL fibroblasts showed that MSC exosomes enhanced proliferation, migration, and collagen synthesis and deposition, which are cellular processes relevant to ACL repair. Further gene set enrichment analysis revealed key pathways mediated by MSC exosomes in enhancing proliferation and migration while reducing matrix degradation of ACL fibroblasts.

**Conclusion:**

The combination of MSC exosomes and fibrin sealant (Exosome+Fibrin) applied to a suture repair enhanced the morphologic and histologic properties of the ACL in a rabbit model, and these improvements could be attributed to the augmented functions of ACL fibroblasts with exosome treatment.

**Clinical Relevance:**

This work supports the use of MSC exosomes in biological augmentation of ACL healing after suture repair.

Surgical treatment of the ruptured anterior cruciate ligament (ACL) has historically been managed with primary repair. This later transitioned to reconstructive options^17^ in light of high failure rates associated with the repairs that became apparent at midterm follow-up^22^ despite good initial results.^25^ Although ACL reconstruction is the current surgical gold standard for treatment,^15^ this is not without significant drawbacks. These include loss of native anatomy, poor proprioception, and donor site morbidity. Additionally, suboptimal outcomes pertaining to graft rupture rates,^26^ reoperation,^19^ and a decline in returning to preinjury level of sporting activity^5^ continue to persist despite advancements in surgical techniques.^20^

This has prompted a renewed interest in primary repair of ACL,^20^ as this offers the prospect of restoring native anatomy, preserving proprioception,^2^ and allowing faster recovery^35^ while avoiding donor site morbidity in ACL reconstruction with autograft use and the inadvertent bone loss from tunnel placement, which can complicate revision surgery. To enhance ACL repair, several studies have explored biological augmentation of ACL healing using biological agents with scaffolds.^14,18,39^ Among the biological agents, there has been an emerging interest in use of mesenchymal stem/stromal cells (MSCs) in combination with scaffolds to augment primary suture repair of the ACL. In one study, MSCs seeded in collagen type I scaffold were found to enhance suture repair of the ACL after transection in a rabbit model, with 33% of specimens showing complete ACL regeneration with a tissue similar to the normal ACL.^14^ Although the mechanisms underlying the therapeutic effects of MSCs remain to be elucidated, coculture studies have shown that MSCs are capable of stimulating functions of ACL fibroblasts, including proliferation, migration, and collagen production, possibly through secretion of trophic factors.^30^ It is now accepted that MSCs mediate tissue repair through the release of extracellular vesicles (EVs). In particular, 50- to 200-nm small EVs (sEVs) that include exosomes carrying a rich cargo of lipids, nucleic acids, proteins, and metabolites have been reported to be therapeutically efficacious in several injuries and diseases.^32^

Selection of an appropriate scaffold for exosome delivery is also of major importance. Ideally, the scaffold should simulate a fibrin clot, creating an environment conducive to healing within the gap between the torn ends of the ACL. Furthermore, the scaffold should preferably be commercially available and clinically used to facilitate clinical translation. As such, Tisseel fibrin sealant is an attractive scaffold of choice for this study.

Herein, we propose a novel Exosome+Fibrin composite gap-bridging strategy to enhance ACL healing. We hypothesize that the addition of Exosome+Fibrin composite to primary suture repair of an ACL midsubstance tear would enhance primary ligament healing in a rabbit model.

## Methods

### Preparation and Characterization of MSC Exosomes

MSC exosomes were purchased from Paracrine Therapeutics Pte Ltd, Singapore. Briefly, the exosomes were derived from an immortalized E1-MYC 16.3 human embryonic stem cell–derived MSC line cultured in Dulbecco's modified Eagle medium (DMEM) containing 10% fetal bovine serum (Thermo Fisher Scientific).^7^ For exosome preparation, the conditioned medium was prepared by growing 80% confluent cells in a chemically defined culture medium composed of DMEM supplemented with 1% nonessential amino acids, 1% glutamine, 1% insulin-transferrin-selenium-X, 1mM sodium pyruvate, 0.05mM β-mercaptoethanol, 5-ng/mL fibroblast growth factor 2 (Thermo Fisher Scientific), and 5-ng/mL platelet-derived growth factor AB (Cytolab). The conditioned medium was size fractionated by tangential flow filtration and concentrated 50× using a membrane with a molecular weight cutoff of 100 kDa (Sartorius).^23^ The exosome preparation was assayed for protein concentration by a Coomassie Plus (Bradford) protein assay kit (Thermo Fisher Scientific), for particle size distribution and concentration by ZetaView (Particle Metrix GmbH), and for CD73/ecto-5′-nucleotidase (NT5E) activity by a PiColorLock Gold Phosphate Detection System (Innova Biosciences), in accordance with the “Minimal Information for Studies of Extracellular Vesicles” guidelines^33,38^ and specifically with the identity and potency metrics proposed for MSC-derived sEV preparations.^16,40^ This protocol has been used for preparation of >100 batches of exosomes, with high batch consistency and reproducibility. In addition to these tests, many earlier batches were analyzed and confirmed to carry exosome-associated markers CD81, ALIX, and TSG101.^43^ For this study, 2 batches of MSC exosomes were used, AC92 and AC117. AC92 had a protein concentration of 1.011 mg/mL, particle concentration of 2.53 × 10^11^/mL, particle modal size of 130.4 nm (Appendix Figure A1, available in the online version of this article), and CD73/NT5E activity of 47.88 ± 0.512 mU/µg of protein (mean ± SD). AC117 had a protein concentration of 1.695 mg/mL, particle concentration of 1.34 × 10^11^/mL, particle modal size of 133.9 nm, and CD73/NT5E activity of 15.81 ± 1.22 mU/µg of protein. The exosomes were 0.22 µm filtered and stored at −20°C until use.

### Animal Study Design and Surgical Procedure

All procedures were performed in accordance with the Institutional Animal Care and Use Committee at our university (protocol No. R17-1274). This study used 12 healthy New Zealand White female rabbits (Envigo), approximately 4 months old with a mean weight of 3.10 ± 0.17 kg (range, 2.9-3.4 kg). The animals were randomly allocated to 2 groups: ACL repairs treated with MSC exosomes and fibrin sealant (Exosome+Fibrin; n = 6) and ACL repairs treated with fibrin sealant (Fibrin; n = 6). The sample size was estimated to be 6 per group, based on our previous study.^42^

**Figure 1.**
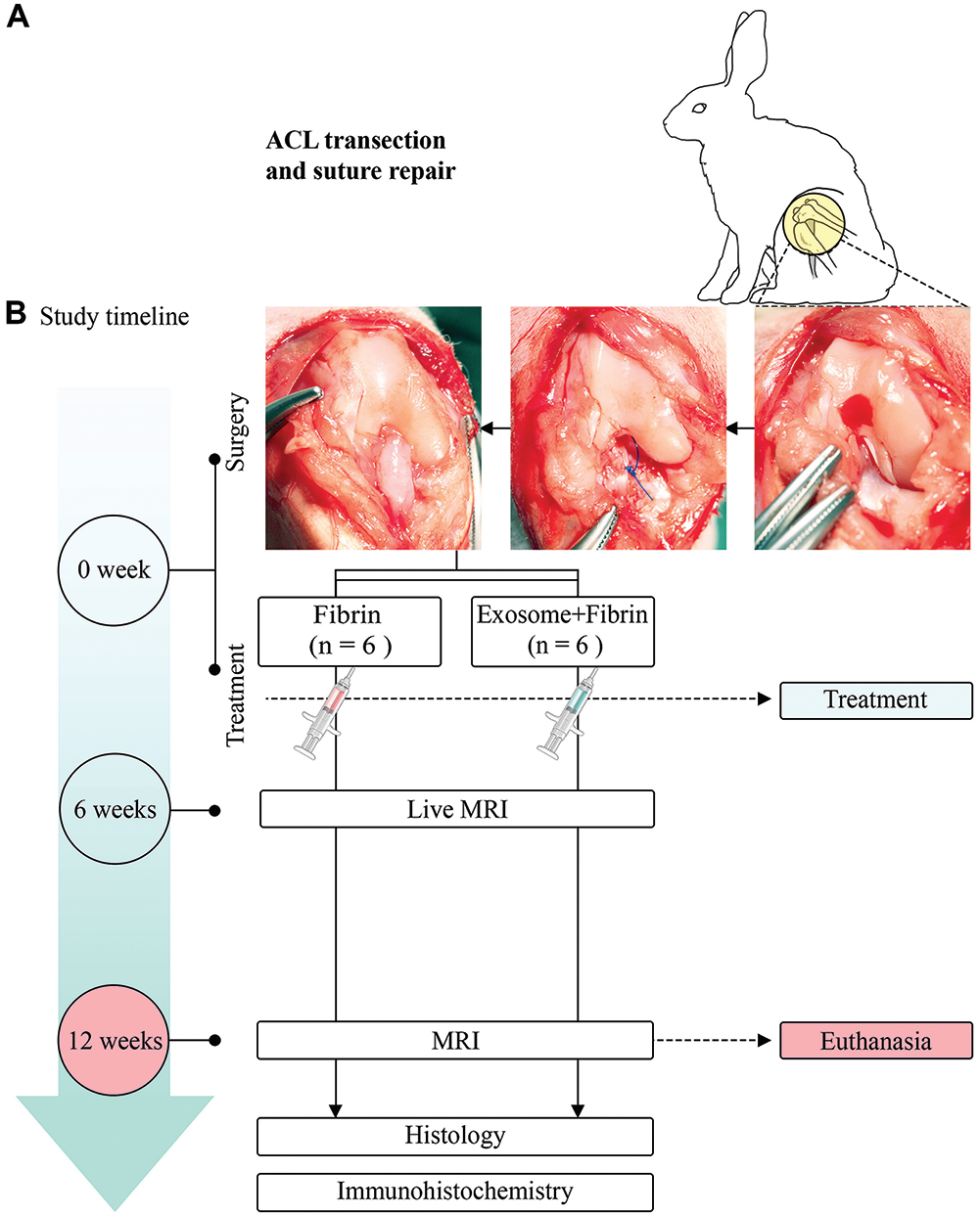
Surgical procedure and study design. (A) Exposure of the intercondylar notch and transection of the ACL at the midsubstance. Approximation of the transected ACL ends and primary suture repair. (B) The rabbits were randomly allocated into 2 groups: ACL repairs treated with MSC exosomes and fibrin sealant (Exosome+Fibrin; n = 6) and ACL repairs treated with fibrin sealant (Fibrin; n = 6). MRI was performed at 6 and 12 weeks, and histologic and immunohistochemical analyses were performed at 12 weeks postoperatively. The sample size for the number of joints is indicated. ACL, anterior cruciate ligament; MRI, magnetic resonance imaging; MSC, mesenchymal stem/stromal cell.

**Figure 2.**
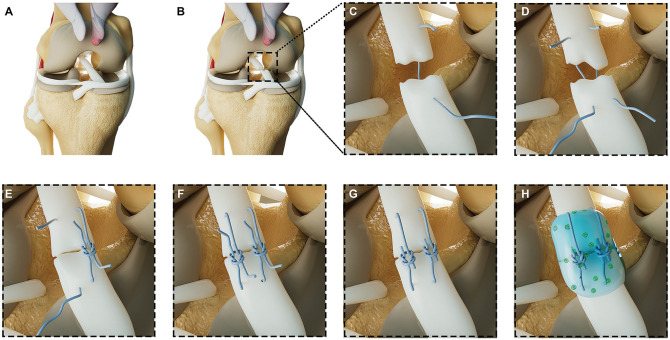
Schematic illustration of the surgical procedure and application of the Exosome+Fibrin composite. (A) Intact ACL preincision. (B) Incision made with surgical blade. (C) First suture placement. (D) Second suture placed after the first suture was pulled to minimize the gap. (E) First suture secured with surgeon's knot. (F) Second suture secured with surgeon's knot. (G) Excess suture trimmed for clean closure and (H) MSC exosomes applied with fibrin sealant for enhanced healing. ACL, anterior cruciate ligament; MSC, mesenchymal stem/stromal cell.

The rabbits were anesthetized with intramuscular ketamine/xylazine (10 mg/kg) and maintained with inhalational isoflurane. The right knee joint was approached through a medial parapatellar incision, and the patella was dislocated laterally to expose the intercondylar notch. The ACL was identified and retracted anteriorly via a dental hook to confirm its integrity. The midpoint of the ACL was then identified as the point between the femoral condyle attachment and the tibial footprint (Figure 1A). A sharp transection of the ACL was performed, and its completeness was confirmed by the inability of the dental hook to retract the ACL. The primary repair was performed with 2 interrupted 5-0 nylon sutures (Ethilon; Ethicon) for approximation of the tear. Tisseel fibrin sealant (Baxter Healthcare) was then applied to the ACL repair in both groups. In the Exosome+Fibrin group, 200 µg of exosomes in 200-µL phosphate-buffered saline (PBS) was mixed with 200 µL of fibrin and applied to the ACL repair, whereas in the Fibrin group, 200-µL PBS was mixed with 200 µL of fibrin and applied to the ACL repair. The fibrin was allowed to set for 5 minutes (Figure 1B). Thereafter, careful hemostasis and layered closure of the arthrotomy and skin were performed. All surgical procedures were performed by an orthopaedic surgeon (K.L.W.). A schematic illustration of the surgical procedure is provided in Figure 2. The rabbits were individually housed, given postoperative carprofen (5 mg/kg) and enrofloxacin (5 mg/kg) for 5 days, and monitored daily for 2 weeks. Animals were allowed to move freely with access to standard food and water. At 12 weeks after surgery, the animals were euthanized by intravenous pentobarbital (150 mg/kg) under general anesthesia, and the joints were harvested for various analyses.

### Magnetic Resonance Imaging

At 6 and 12 weeks after surgery, animals were anesthetized and placed supine on the imaging bed. Live magnetic resonance imaging (MRI) of the knee joints was performed with a standard knee coil on a Magnetom Skyra 3T scanner (Siemens). The scanning sequence parameters were as follows: for the T2-weighted sagittal position, a repetition time (TR) of 1660 milliseconds, an echo time (TE) of 23.6 milliseconds, a layer thickness of 2 mm, and a field of view (FOV) of 120 mm; for the proton density–weighted coronal position, a TR/TE of 3460/30 milliseconds, a layer thickness of 2 mm, and an FOV of 100 mm.

At 12 weeks, a high-resolution ex vivo 7T MRI scan of the dissected knees was performed on a Magnetom ClinScan syngo 7T scanner (Siemens) using the following parameters: for the T2-weighted turbo spin echo sagittal position, a TR/TE of 1430/37 milliseconds, a layer thickness of 0.5 mm, and an FOV of 80 mm; for the T2-weighted turbo spin echo coronal position, a TR/TE of 1290/37 milliseconds, a layer thickness of 0.5 mm, and an FOV of 60 mm. The MRI scans were viewed with a Digital Imaging and Communications in Medicine viewer (RadiAnt Version 2020; Medixant).

Using 3T MRI sagittal sequences, the ligaments were graded as completely healed if there was complete restoration of fiber continuity, partially healed if there was partial restoration of fiber continuity, or torn if there was no observable restoration. The grading was performed by K.L.W. and S.Z. in a blinded fashion. Using 7T MRI sequences, the maturation of ligaments was graded by the intensity as previously described^37^: hypointense if the intensity of the repaired ACL was similar to the posterior cruciate ligament (PCL), isointense if >50% of the ACL had a similar signal as the PCL, and hyperintense if <50% of the ACL had the same intensity as the PCL. The grading was performed by G.W.L. and S.Z. in a blinded fashion.

### Histologic and Immunohistochemical Staining Analyses

Rabbit knees were dissected and fixed in 10% (v/v) neutral buffered formalin for 1 week and decalcified in 30% (v/v) formic acid for 3 weeks. The specimens were dehydrated, paraffin embedded, and sectioned sagittally at 5-µm thickness. Sections were stained with hematoxylin and eosin to assess the general morphology and with Masson trichrome to observe the ligament (green). Immunohistochemical staining for collagen I (1:1000; Sigma) and collagen III (1:500; Novus Biologicals) was performed with the UltraVision Quanto Detection System (Thermo Fisher Scientific). Digital images were taken at 12.5× magnification by an inverted microscope (IX70; Olympus) and merged via Adobe Photoshop CS6 (Adobe Systems).

### Isolation and Culture of ACL Fibroblasts

Primary ACL fibroblasts were harvested from the ACLs of 6 healthy New Zealand White female rabbits. Tissues were washed 3 times with PBS containing 1% penicillin-streptomycin (Thermo Fisher Scientific) and digested in 0.2% (w/v) collagenase I (Worthington) for 4 hours at 37°C. The cells were then passed through a 40-µm cell strainer (Corning) to disperse into single cells before being seeded at a density of 2 × 10^4^ cells/cm^2^. Cells were cultured in DMEM/F12 supplemented with 10% fetal bovine serum and 1% penicillin-streptomycin under a humidified atmosphere of 5% CO_2_ at 37°C. The medium was changed every alternate day. Upon confluence, cells were subcultured to passage 2 (P2) for subsequent in vitro experiments.

### Cell Metabolic Activity and Proliferation

P2 ACL fibroblasts were seeded at 5000 cells/well in 96-well plate and treated with 1, 5, or 10 µg/mL of exosomes or PBS. At 4, 24, 48, and 72 hours, cell metabolic activity was measured by an MTS assay kit (3-[4,5-dimethylthiazol-2-yl]-5-[3-carboxymethoxyphenyl]-2-[4-sulfophenyl]-2H -tetrazolium; Promega) following the manufacturer's instructions. Briefly, 20 µL of MTS reagent was added into each well and incubated at 37°C for 2 hours. Absorbance readings were then recorded at 490 nm and 650 nm (reference) using a microplate reader (Spark; Tecan). Total DNA content that is reflective of the cell number was measured as previously described.^34^ Briefly, cells were lysed by adding 30 μL of lysis buffer (Thermo Fisher Scientific) to each well, and the DNA concentration was measured by the Quant-iT Picogreen dsDNA assay kit (Thermo Fisher Scientific) following the manufacturer's instructions. Fluorescence readings were taken at an excitation of 480 nm and emission of 520 nm using the microplate reader. DNA concentration was calculated per the DNA reference standard.

### Cell Migration

The migration of ACL fibroblasts in response to exosome treatment was assessed using a Transwell system.^44^ Briefly, 1.5 × 10^4^ cells in 300 µL of low-serum culture medium (DMEM/F12 supplemented with 0.5% fetal bovine serum and 1% penicillin-streptomycin) were placed in the Transwell insert, and 1, 5, or 10 µg/mL of exosomes or PBS was added to the bottom well. After 4 hours, the upper surface of the Transwell inserts was swabbed free of cells. Cells on the underside of the insert were fixed with 10% formalin (v/v) for 1 hour and stained with hematoxylin and eosin. The migrated cells were counted at 5 randomly selected fields at 100× magnification using ImageJ software (National Institutes of Health) and expressed as mean number of cells per high-power field.

### Collagen and DNA Quantification

Total collagen deposited and soluble collagen released into the culture media by the ACL fibroblasts were measured by the Sircol collagen assay kit (Biocolor) following the manufacturer's instructions. At 4, 24, 48, and 72 hours, the cells and culture supernatants were harvested. The cells were divided for DNA and collagen quantification assays. Total DNA content was measured by the Quant-iT Picogreen dsDNA assay kit. The remaining cells were digested overnight at 4°C using 0.1 mg/mL of pepsin in 0.5M acetic acid. The isolation/concentration reagent provided in the kit was then added to the culture supernatants and washed with the acid-salt wash reagent to precipitate the collagen. The precipitated collagen from the culture supernatants and digested cell lysates was added with the Sircol dye reagent and incubated for 30 minutes. Thereafter, the samples were centrifuged to pellet the collagen-dye complex, and excessive dye was removed by washing with ice-cold acid-wash reagent. Finally, alkali reagent was added to dissolve the bound dye, and the absorbance reading was taken at 556 nm using the microplate reader. The collagen concentration was calculated per the collagen reference standard, normalized with the total DNA, and presented as total collagen/DNA and soluble collagen/DNA.

### RNA Sequencing

Total RNA was isolated from primary rabbit ACL fibroblasts treated with 10 μg/mL of exosomes or a similar volume of PBS for 24 hours via a Maxwell RSC simplyRNA Tissue Kit (Promega). RNA was quantified by a Qubit RNA Broad Range Assay Kit and fluorometer (Thermo Fisher Scientific) and assessed for degradation based on RNA integrity number (RIN) by the Agilent 4150/4200 TapeStation (Agilent). The Zymo Seq RiboFree Total RNA Library Kit was used to assess transcript abundance following standard instructions from the manufacturer. Briefly, 150 ng of total RNA with an RNA integrity number >8 was reverse transcribed to cDNA using a random hexamer primer before rRNA depletion. Next, partial P7 and P5 adapters were ligated to the rRNA-depleted cDNA, amplified, and indexed before purification. The final libraries were quantified by a Qubit DNA Broad Range Assay Kit, and the mean fragment size was determined by the 4150/4200 TapeStation (Agilent). All final libraries were then pooled before circularization for MGI sequencer compatibility. Finally, the pooled library was sequenced on a DNBSEQ-T7 Sequencer (MGI) using paired-end 150–base pair sequencing chemistry.

### RNA Sequencing Analysis

Libraries were demultiplexed according to unique index pairs. Adapter sequences were then trimmed using trimmomatic (Version 0.36)^4^ in paired-end mode with the options MAXINFO:35:0.5 MINLEN:35. Trimmed reads were aligned to the *Oryctolagus cuniculus* OryCun2.0 using STAR (Version 2.7.9a)^12^ with the options *−outFilterType BySJout –outFilterMultimapNmax 20 –alignSJoverhangMin 8 –alignSJDBoverhangMin 1 –outFilterMismatchNmax 999 –alignIntronMin 20 –alignIntronMax 1000000 –alignMatesGapMax 1000000* in paired-end, single-pass mode. Only unique alignments were retained for counting. Counts were calculated at the gene level by the FeatureCounts module from subread (Version 2.0.3), with the options *-O -s 2 -J -T 8 -p -R CORE -G*. The Ensembl Release 111 *Oryctolagus cuniculus* OryCun2.0 GTF was used as annotation to prepare STAR indexes and for FeatureCounts. For sample groups, the design for the model was specified as *~stimulus* (Exosome or PBS treatment). Gene set enrichment analyses were run using fgsea (Version 1.24.0) with 10^5^ permutations against the following gene set databases: *Homo sapiens* MSigDB (msigdbr Version 7.5.1), Kyoto Encyclopaedia of Genes and Genomes (KEGG),^21^ Reactome,^11^ and Hallmark.^24^ Visualizations were generated using ggplot2 R packages and GraphPad Prism.

### Statistical Analysis

All quantitative data were presented as mean and standard deviation (SD). Data distribution was checked by tests of normality. Differences between groups were determined as follows: for normally distributed data, by Student *t* test or 1-way analysis of variance, followed by Bonferroni post hoc test; for nonnormally distributed data, by Mann-Whitney *U* test or Kruskal-Wallis test, followed by Dunn-Bonferroni post hoc test. Statistical analysis was performed with SPSS Statistics Version 26.0 (IBM) with significance set as *P* < .05.

## Results

### MRI Evaluation of ACL Repair

**Figure 3.**
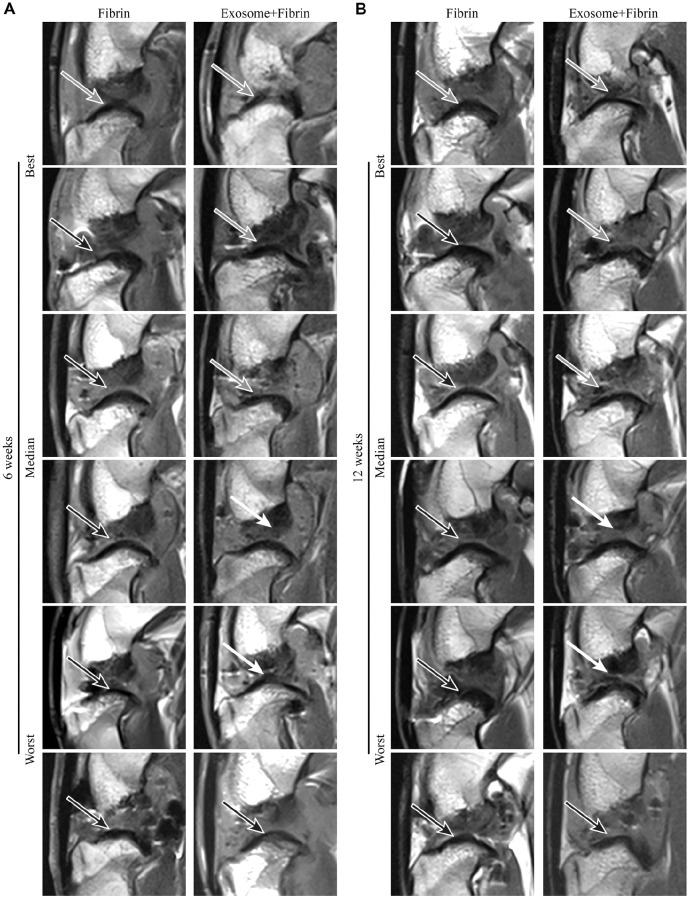
3T MRI analysis of ACL repair. (A, B) 3T MRI of the rabbit knees was performed at 6 and 12 weeks postoperatively. Representative images of the ACLs achieving the best, median, and worst repair (n = 6). Completely healed ACL, gray arrow; partially healed, white arrow; torn, black arrow. ACL, anterior cruciate ligament; MRI, magnetic resonance imaging.

At 6 and 12 weeks, live MRI scans were performed. Samples were ranked best, median, or worst repair (Figure 3). At 6 weeks, 3T MRI grading showed general improvements in ACL healing in the Exosome+Fibrin group as compared with the Fibrin group. Of the 6 ACLs in the Exosome+Fibrin group, 3 showed complete healing, 2 showed partial healing, and 1 showed no evidence of healing and remained torn. In contrast, only 1 ACL in the Fibrin group showed complete healing, and the remaining 5 ACLs had no evidence of healing and remained torn (Figure 3A). At 12 weeks, the 3T MRI grading of the ACL repair remained unchanged. Of the 6 ACLs repaired in the Exosome+Fibrin group, 3 showed complete healing, 2 showed partial healing, and 1 showed no evidence of healing and remained torn. On the contrary, only 1 ACL in the Fibrin group showed complete healing, and the remaining 5 ACLs remained torn (Figure 3B). The results are summarized in Table 1.

**Table 1:** ACL Repairs^*a*^

		Fibrin					Exosome+Fibrin			
		MRI					MRI			
Rank	ID	3T, 6 wk	3T, 12 wk	7T, 12 wk	Histology, 12 wk	ID	3T, 6 wk	3T, 12 wk	7T, 12 wk	Histology, 12 wk
1	1076	Complete	Complete	Isointense	Complete	1043	Complete	Complete	Isointense	Complete
2	1203	Torn	Torn	Hyperintense	Torn	0907	Complete	Complete	Isointense	Complete
3	1062	Torn	Torn	Hyperintense	Torn	1032	Complete	Complete	Isointense	Complete
4	1104	Torn	Torn	Hyperintense	Torn	0977	Partial	Partial	Isointense	Partial
5	1105	Torn	Torn	Hyperintense	Torn	1020	Partial	Partial	Hyperintense	Torn
6	1114	Torn	Torn	Hyperintense	Torn	0941	Torn	Torn	Hyperintense	Torn

a ACL, anterior cruciate ligament; MRI, magnetic resonance imaging.

**Figure 4.**
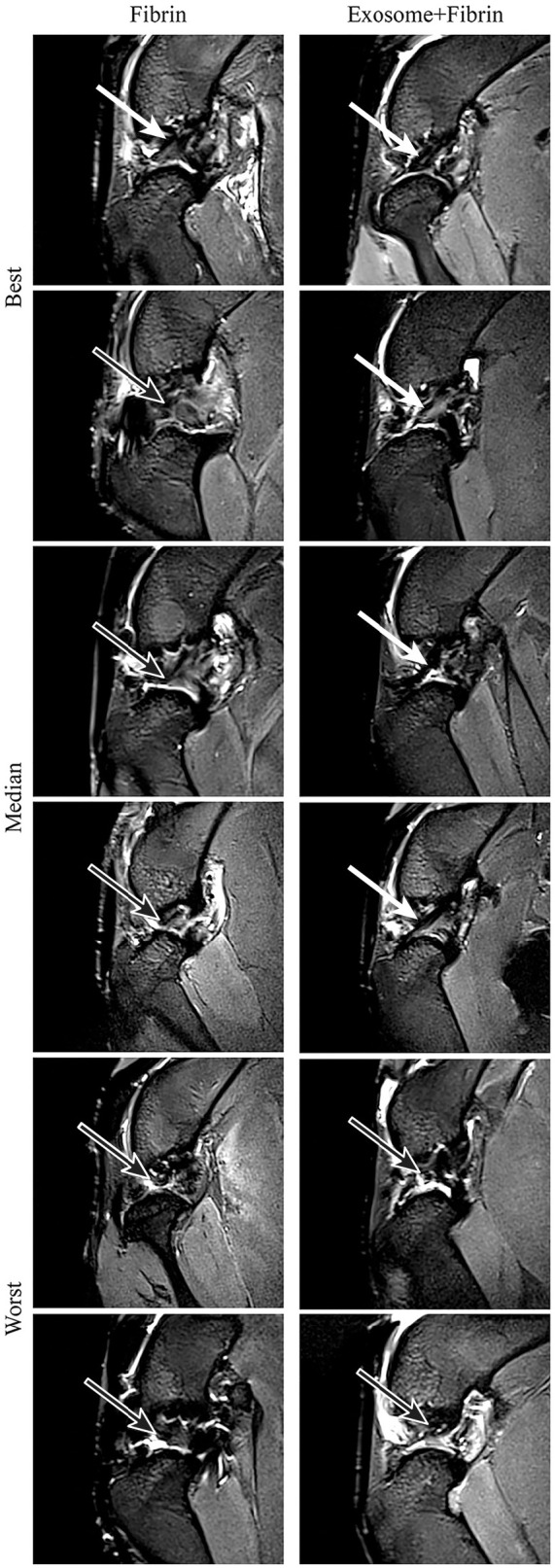
7T MRI analysis of ACL repair. 7T MRI of the rabbit knees was performed at 12 weeks postoperatively. Representative images of the ACLs presented at the sagittal view show the best, median, and worst repair (n = 6). Isointense ACL, white arrow; hyperintense, black arrow. ACL, anterior cruciate ligament; MRI, magnetic resonance imaging.

Using 7T MRI sagittal sequences, the intensity of ACLs at 12 weeks was examined to evaluate the maturation of the ligaments (Figure 4). Among the Exosome+Fibrin–treated ACLs, 4 ACLs were isointense and 2 ACLs were hyperintense. On the contrary, 1 ACL was isointense and the remaining 5 ACLs appeared hyperintense among the Fibrin-treated ACLs. The results are summarized in Table 1.

### Histologic Evaluation of ACL Repair

**Figure 5.**
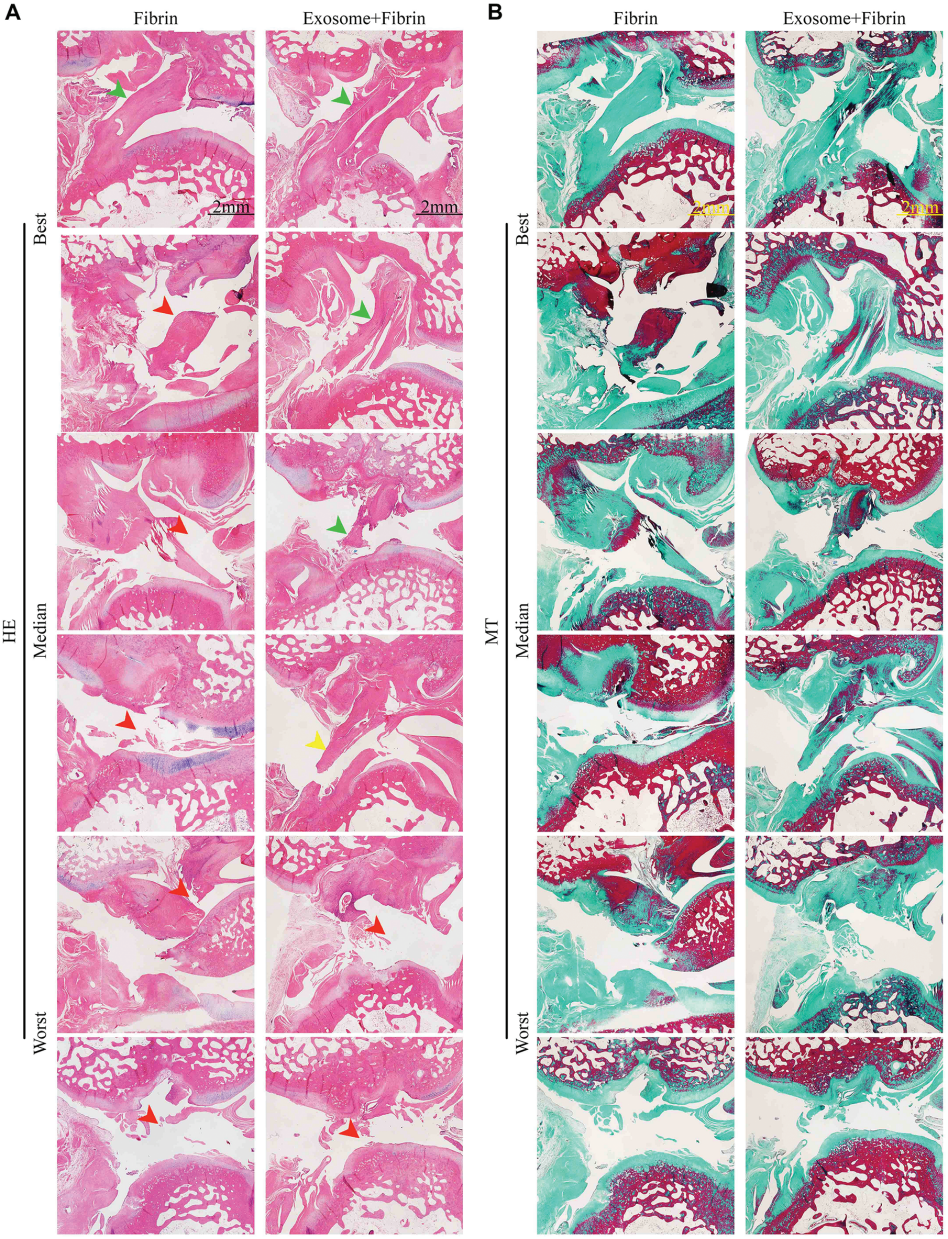
Histologic assessment of ACL repair. (A, B) Histologic analysis by hematoxylin and eosin (HE) and Masson trichrome (MT) staining. Representative images of the ACLs achieving the best, median, and worst ACL repair (n = 6). Completely healed ACL, green arrowhead; partially healed, yellow arrowhead; torn, red arrowhead. Scale bar: 2 mm. ACL, anterior cruciate ligament.

**Figure 6.**
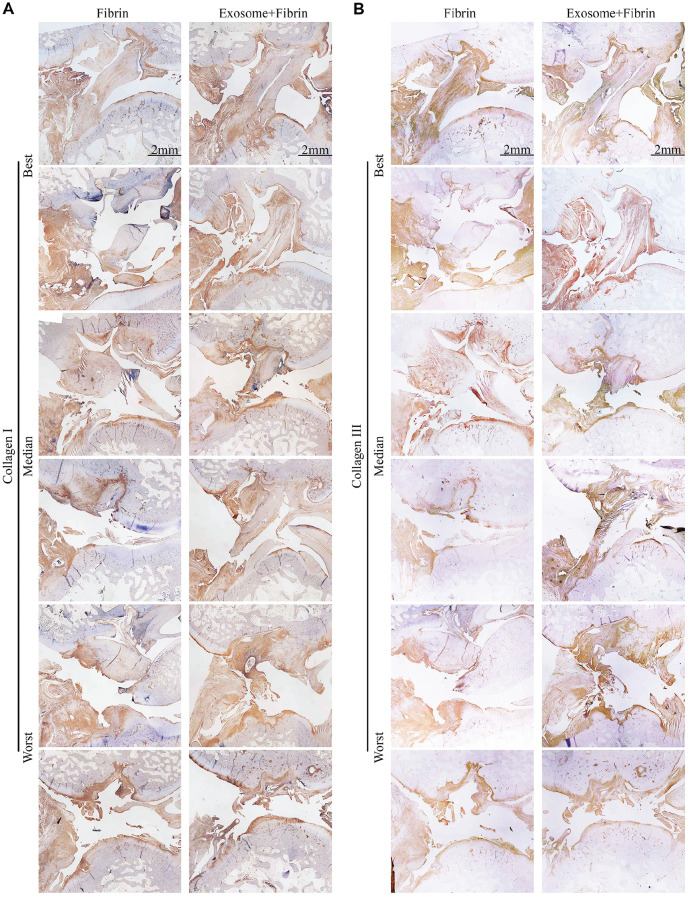
Ligament-specific matrix expression of ACL repair. (A, B) Immunohistochemical staining for collagen I and collagen III. Representative images of the ACLs achieving the best, median, and worst ACL repair (n = 6). Scale bar: 2 mm. ACL, anterior cruciate ligament.

MRI findings correlated fairly well with the histologic findings at 12 weeks (Table 1). Of the 6 repaired ACLs in the Exosome+Fibrin group, 4 ACLs (3 completely healed and 1 partially healed) showed newly formed ligamentous tissue with abundant collagen deposition, specifically, a high amount of collagen I and a relatively lower amount of collagen III (Figures 5 and 6). The remaining 2 ACLs showed no evidence of healing and remained torn. In contrast, only 1 ACL in the Fibrin group had complete healing, with newly formed ligamentous tissue showing abundant collagen deposition but with a high amount of collagen III and a relatively lower amount of collagen I. The remaining 5 ACLs remained torn. The normal ligament consists of 90% collagen I, 9% collagen III, and other proteoglycans.^1^ It was interesting to note that collagen I was more prominent in comparison with collagen III in the Exosome+Fibrin–treated ACLs, which is consistent with the matrix composition of the native ligament.

### Effects of MSC Exosomes on ACL Fibroblasts

**Figure 7.**
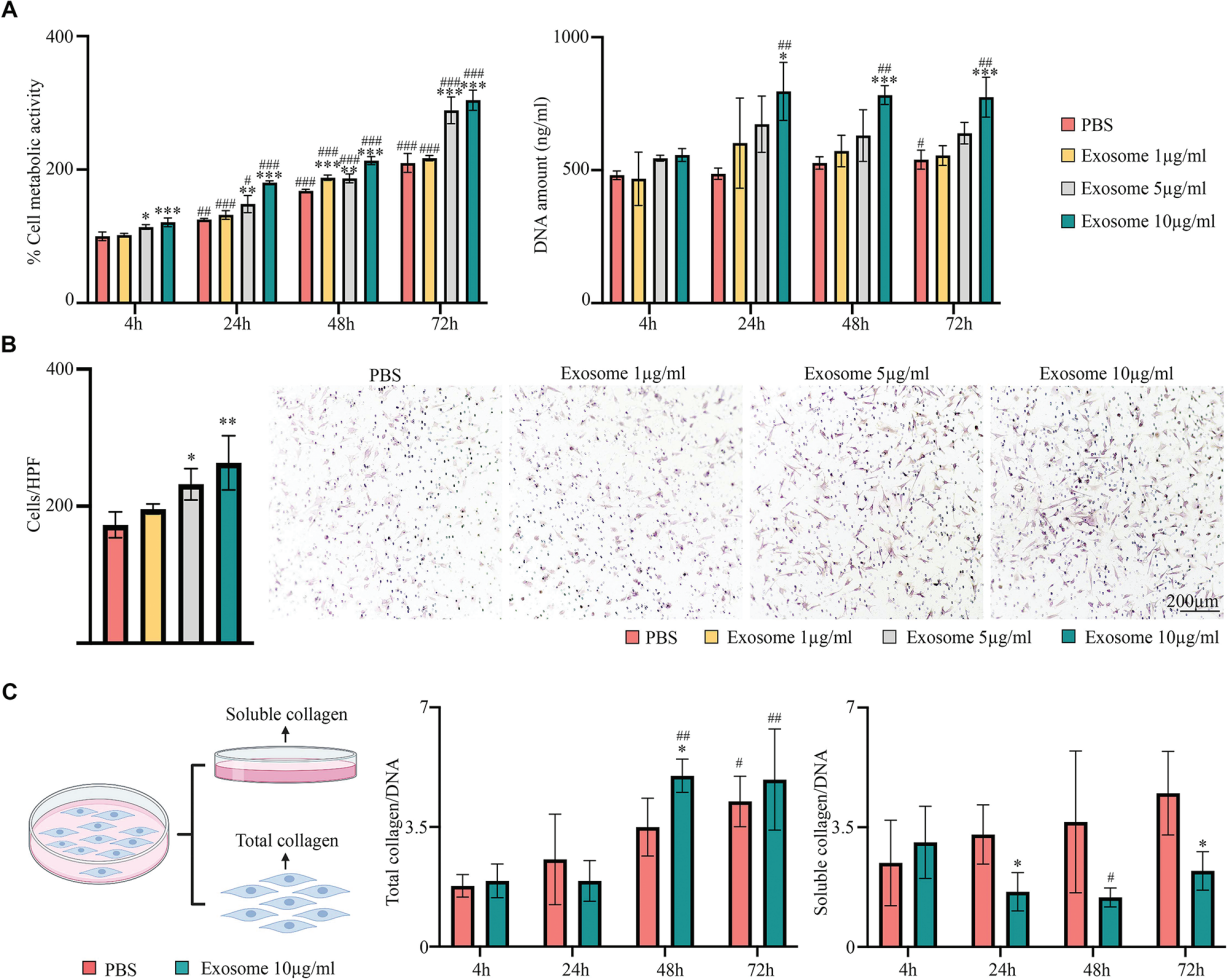
Effects of MSC exosomes on functions of ACL fibroblasts. (A) MTS metabolic assay (left) and DNA assay (right) show potent dose- and time-dependent effects of MSC exosomes on metabolic activity and proliferation of ACL fibroblasts. (B) Transwell migration assay demonstrates dose-dependent effect of MSC exosomes on migration of ACL fibroblasts. (C) Quantification of total collagen deposited and soluble collagen released into the culture supernatants by ACL fibroblasts indicates that MSC exosomes significantly enhance collagen synthesis and deposition while reducing the loss of collagen into the culture media. Data are presented as mean ± SD. Compared with the PBS group within the same time point (n = 4): *P < .05. **P < .01. ***P < .001. Compared with 4 hours within the same treatment group (n = 4): ^#^*P* < .05. ^##^*P* < .01. ^###^*P* < .001. Scale bar: 200 µm. ACL, anterior cruciate ligament; HPF, high-power field; MSC, mesenchymal stem/stromal cell; PBS, phosphate-buffered saline.

To explore the cellular processes activated by MSC exosomes during ACL repair, cell culture studies utilizing rabbit ACL fibroblasts were performed. We found that MSC exosomes significantly enhanced the cell metabolic activity as early as 4 hours, with significant enhancement at 10 µg/mL of exosomes as compared with PBS control (*P* < .001). From 4 to 72 hours, exosome treatment significantly increased the metabolic activity of ACL fibroblasts in a dose- and time-dependent manner (Figure 7A). Consistent with the increase in metabolic activity, the DNA content reflective of the cell number increased with rising concentrations of MSC exosomes. The treatment with 10 µg/mL of exosomes significantly increased the cell number as compared with PBS control as early as 24 hours (*P* = .014). By the end of 72 hours, the number of cells was ~44% higher than those treated with PBS (*P* < .001).

In addition, we observed that ACL fibroblasts exhibited increased migratory activity with a higher concentration of MSC exosomes (Figure 7B). Relative to the number of migrated cells with PBS treatment, the number of migrated cells was ~34% higher with 5 μg/mL of exosomes (*P* = .034) and ~52% higher with 10 μg/mL of exosomes (*P* = .002). Thus, MSC exosomes have the potential to elicit a rapid migration of ACL fibroblasts into the defect site during ACL repair.

Quantification of collagen further showed that MSC exosomes enhanced collagen synthesis and deposition by ACL fibroblasts, while reducing their loss of collagen into the media (Figure 7C). At 48 hours after exosome treatment, the level of collagen deposited by ACL fibroblasts was significantly increased in comparison with those treated with PBS (*P* = .022). In parallel, exosome treatment significantly reduced the level of soluble collagen released into the media by ~50% at 24 hours (*P* = .018) and 72 hours (*P* = .015). These findings suggest effective collagen synthesis and retention by the ACL fibroblasts with exosome treatment, as also evidenced by rich collagen deposition in the newly formed ligamentous tissue observed in our animal model.

### Gene Set Enrichment Analysis

**Figure 8.**
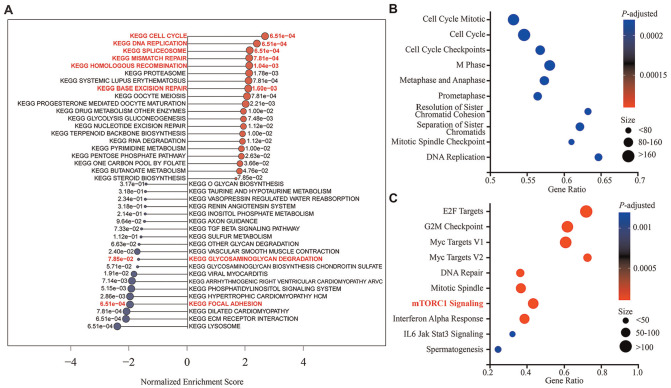
Gene set enrichment analysis of primary ACL fibroblasts, comparing MSC exosome and PBS treatment groups. (A) KEGG pathway analysis highlights key pathways for cell proliferation, migration, and matrix degradation, with positive scores for events such as cell cycle and DNA replication and negative scores for pathways including focal adhesion and glycosaminoglycan degradation. (B) Reactome analysis reveals the pivotal roles of cell cycle progression, including the various mitotic phases. (C) Hallmark analysis demonstrates upregulation of signaling pathways such as mTORC1 signaling, which plays an important role in regulating cell growth, proliferation, and extracellular matrix biosynthesis. n = 3 per group. ACL, anterior cruciate ligament; KEGG, Kyoto Encyclopaedia of Genes and Genomes; MSC, mesenchymal stem/stromal cell; mTORC1, mammalian target of rapamycin complex 1; PBS, phosphate-buffered saline.

After confirmation of the effects of MSC exosomes on ACL repair in vitro and in vivo, RNA sequencing was performed in primary ACL fibroblasts treated with exosomes or PBS to elucidate the possible mechanisms underlying the effects of MSC exosomes in ACL repair (Figure 8). Gene set enrichment analysis revealed several pathways with significant enrichment scores. KEGG pathway analysis identified positive normalized enrichment scores (NES) for cell cycle (NES = 2.54), DNA replication (NES = 2.26), and spliceosome (NES = 2.03), which are crucial for cell division, genetic information replication, and RNA processing, respectively, suggesting their involvement in promoting proliferation of ACL fibroblasts (Figure 8A). Pathways related to DNA repair mechanisms, such as mismatch repair (NES = 2.00), homologous recombination (NES = 2.00), and base excision repair (NES = 1.97), were also positively enriched, implicating their involvement in maintaining genomic stability and integrity during cell proliferation. On the contrary, the focal adhesion pathway exhibited a negative enrichment score (NES = −1.85), suggesting potential alterations in focal adhesion dynamics that might influence the adhesive and migratory properties of ACL fibroblasts. Furthermore, the glycosaminoglycan degradation pathway showed a negative enrichment score (NES = −1.64), suggesting reduced degradation of glycosaminoglycan, a major extracellular matrix (ECM) component of ACL, crucial for its structural integrity and tissue elasticity.

Further elucidation through Reactome and Hallmark pathway analyses reinforced these findings. The top upregulated Reactome pathways included cell cycle mitotic, cell cycle, cell cycle checkpoints, M phase, metaphase and anaphase, prometaphase, resolution of sister chromatids, separation of sister chromatids, mitotic spindle checkpoint, and DNA replication (Figure 8B), highlighting their fundamental roles in cell cycle progression and chromosomal segregation. The Hallmark pathways showed significant upregulation of E2F targets, G2M checkpoint, Myc targets V1, Myc targets V2, DNA repair, mitotic spindle, mammalian target of rapamycin complex 1 (mTORC1) signaling, interferon alpha response, IL6 Jak Stat3 signaling, and spermatogenesis. Pathways such as E2F targets, G2M checkpoint, Myc targets, DNA repair, and mitotic spindle are directly implicated in cell cycle regulation and cellular proliferation processes, while mTORC1 signaling plays a critical role in regulating cell growth, proliferation, and ECM biosynthesis (Figure 8C).

Collectively, the pathways identified through KEGG, Reactome, and Hallmark analyses illuminate key regulatory mechanisms underpinning enhanced proliferation, migration, and matrix retention of ACL fibroblasts, providing profound insights into the molecular processes driving these cellular activities.

## Discussion

We have demonstrated for the first time that the addition of MSC exosomes and fibrin sealant (Exosome+Fibrin composite) to the suture repair of an ACL midsubstance tear enhanced primary ligament healing in a rabbit model. This principal finding is supported by comprehensive MRI, histologic, and immunohistochemical analyses that demonstrated improved morphologic and histologic properties of the repaired ACL with Exosome+Fibrin treatment. Based on 3T MRI analysis, 5 of 6 Exosome+Fibrin–treated ACLs were completely or partially healed, as opposed to 5 of 6 Fibrin-treated ACLs that appeared torn at 6 and 12 weeks postoperatively. By 7T MRI analysis, 4 of 6 Exosome+Fibrin–treated ACLs were isointense, as compared with 5 of 6 Fibrin-treated ACLs that were hyperintense at 12 weeks. This suggests improved remodeling and maturation of the repaired ACLs with Exosome+Fibrin treatment. Histologically, the Exosome+Fibrin–treated ACLs displayed more organized collagen fibers and enhanced collagen deposition, with more collagen I and relatively less collagen III in the best-ranked samples, which were not observed in the Fibrin-treated ACLs. This transition in matrix composition suggests enhanced tissue remodeling toward a more mature ACL with Exosome+Fibrin treatment.

ACL tears occur most commonly in the midsubstance location, accounting for >50% of all ACL injuries.^36^ Despite this high incidence, effective treatment strategies to promote ACL healing are lacking. Unlike proximal ACL tears, ACL repair of midsubstance tears is particularly challenging owing to poor vascularity and has consequently yielded poor postoperative results.^31^ Although the development and application of innovative fixative devices have improved primary ACL repair, some inherent limitations remain. For instance, scaffold supplementation with autologous whole blood requires an additional procedure in blood collection from the patient.^27^ Other studies have explored scaffold supplementation with autologous MSCs^14^ or ligament cells.^39^ However, these approaches require harvesting the cells and expanding and reimplanting them, with significant operational costs and challenges in maintaining the sterility of culture conditions and the viability of cells required for transplantation.

In this study, we showed that MSC exosomes derived from an immortalized E1-MYC 16.3 human MSC line could be supplemented to fibrin sealant to augment ACL healing after suture repair. These cells grow faster and have increased telomerase activity while retaining the parental karyotype, thus providing an unlimited supply of cells for a scalable production of exosomes in a consistent and reproducible manner required for clinical translation.^7^ Additionally, these MSC exosomes are immune privileged like their parental cells and did not elicit any observable adverse responses from the immunocompetent animals used here and in previous studies,^41,42,45^ suggesting the potential allogenic application of these MSC exosomes. Furthermore, these MSC exosomes can be stored at −20°C for up to 6 months without the loss of bioactivity, making them attractive for off-the-shelf applications.

The healing of the injured ACL is primarily achieved by fibroblasts, which migrate from the ACL into the wound site.^29^ Once these cells arrive at the wound site, key cellular processes such as cell proliferation and collagen synthesis result in the formation of a fibrovascular scar, which then remodels into tissue with features closer to those of the normal ligament.^28^ We therefore tested the MSC exosomes on ACL fibroblasts and demonstrated that MSC exosomes could enhance proliferation, migration, as well as collagen synthesis and retention of ACL fibroblasts to promote ACL repair. These anabolic effects of MSC exosomes on ACL fibroblasts are in agreement with previous studies that reported prolific effects of MSC exosomes on proliferation, migration, and matrix synthesis of numerous cell types, including chondrocytes,^44^ endothelial cells,^9^ and periodontal ligament cells.^8^

Consistent with these observations, gene set enrichment analysis utilizing the KEGG, Reactome, and Hallmark databases revealed positive enrichment in pathways associated with cell cycle and DNA replication, suggesting that MSC exosomes could accelerate cell division to promote proliferation of ACL fibroblasts for effective ACL repair. On the other hand, negative enrichment was observed in pathways such as focal adhesion and glycosaminoglycan degradation, which are implicated in cell adhesion, migration, and ECM homeostasis. Focal adhesions play pivotal roles in substrate adhesion, cell spreading, and migration.^6^ By modulating the focal adhesions, MSC exosomes potentially regulate the interactions of ACL fibroblasts with the ECM to influence the adhesive and migratory dynamics of ACL fibroblasts during ACL repair. Restoring ECM homeostasis is crucial for effective tissue healing following injury.

On this note, we found that MSC exosomes are capable of promoting matrix synthesis while suppressing matrix degradation to protect against matrix loss during ACL injury, thereby restoring tissue integrity and stability in ACL repair. The diverse effects of MSC exosomes were mediated through multiple signaling pathways. Among these, mTORC1 signaling is well known for regulating cell growth and proliferation.^3^ In human ACL fibroblasts, rapamycin inhibition of mTORC1 decreased collagen synthesis and deposition while increasing collagen degradation.^13^ Similarly, inhibition of mTORC1 inhibited collagen I production and impaired tenogenesis of MSCs.^10^ Therefore, MSC exosomes could restore matrix homeostasis of ACL fibroblasts by enhancing matrix synthesis while inhibiting matrix degradation through the mTORC1 signaling pathway.

Our results demonstrated the feasibility and efficacy of an Exosome+Fibrin composite gap-bridging strategy to enhance ACL healing in a rabbit model. However, this study has some limitations. The current animal model may not fully replicate the ligament injury that occurs in an acute ACL tear. Although there was general improvement in ACL healing with Exosome+Fibrin treatment, tissue remodeling appeared incomplete at 12 weeks. Longer-term observations may be needed to determine whether the ACL repair and remodeling will progress to maturation. Studies using male rabbits may be considered to determine any sex differences. Also, we did not perform biomechanical testing of the rabbit ACLs. Further investigation in a larger animal model (pig) would be necessary to gain insights into the biomechanical properties and functional competency of the repaired ACLs required for clinical translation.^18^

## Conclusion

We show that the addition of MSC exosomes and fibrin sealant (Exosome+Fibrin composite) to the suture repair of an ACL midsubstance tear enhanced primary ligament healing with morphologic and histologic improvements in a rabbit model. This augmented ACL healing could be attributed to exosome-mediated enhancement of ACL fibroblast proliferation, migration, and collagen production. Our study highlights a newly developed gap-bridging strategy utilizing MSC exosomes as an off-the-shelf and cell-free therapeutic to enhance primary ACL suture repair.

## Supplemental Material

sj-pdf-1-ajs-10.1177_03635465241313142 – Supplemental material for Mesenchymal Stem Cell Exosome and Fibrin Sealant Composite Enhances Rabbit Anterior Cruciate Ligament Repair Supplemental material, sj-pdf-1-ajs-10.1177_03635465241313142 for Mesenchymal Stem Cell Exosome and Fibrin Sealant Composite Enhances Rabbit Anterior Cruciate Ligament Repair by Keng Lin Wong, Kristeen Ye Wen Teo, Gin Way Law, Shipin Zhang, Tianqi Wang, Hassan Afizah, Chee Jian Pua, Barry Wei Loong Tan, James Hoi Po Hui and Wei Seong Toh in The American Journal of Sports Medicine
