# Supplementary material for: Mesenchymal Stem Cell Exosome and Fibrin Sealant Composite Enhances Rabbit Anterior Cruciate Ligament Repair
Source: Am J Sports Med. 2025 Feb 21;53(4):871–84. doi: 10.1177/03635465241313142 (PMC11894873; doi:10.1177/03635465241313142)
Supplement: sj-pdf-1-ajs-10.1177_03635465241313142 – Supplemental material for Mesenchymal Stem Cell Exosome and Fibrin Sealant Composite Enhances Rabbit Anterior Cruciate Ligament Repair [file sj-pdf-1-ajs-10.1177_03635465241313142.pdf]

Mesenchymal stem cell exosomes and fibrin sealant composite enhances rabbit anterior cruciate ligament repair

Appendix

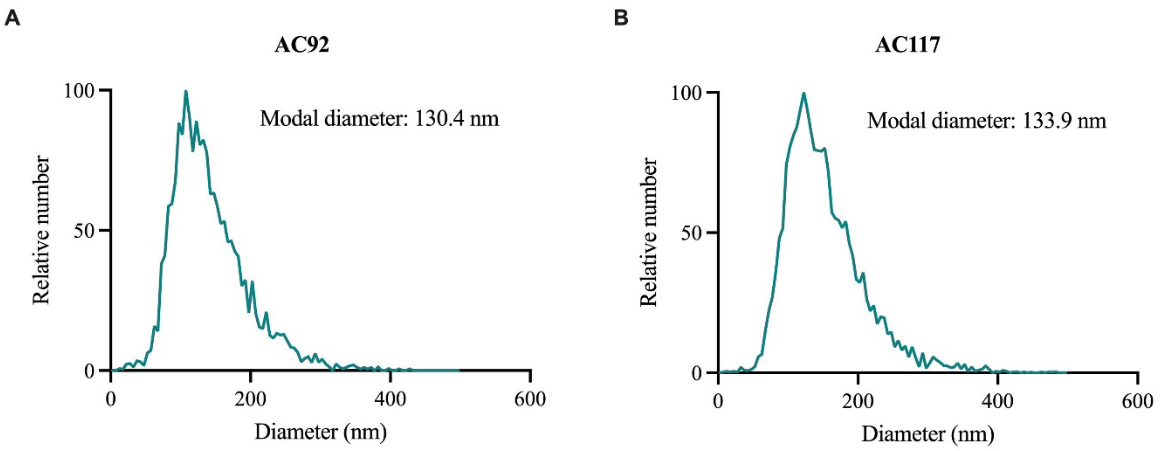

Figure A1. Size distribution of MSC exosomes (A) AC92 and (B) AC117 using nanoparticle tracking analysis.
